# Rate of sequence divergence under constant selection

**DOI:** 10.1186/1745-6150-5-5

**Published:** 2010-01-21

**Authors:** Alexey S Kondrashov, Inna S Povolotskaya, Dmitry N Ivankov, Fyodor A Kondrashov

**Affiliations:** 1 Life Sciences Institute, Department of Ecology and Evolutionary Biology, University of Michigan, Ann Arbor, MI 48109, USA; 2 Bioinformatics and Genomics Programme, Centre for Genomic Regulation, C/Dr Aiguader 88, Barcelona Biomedical Research Park Building, 08003 Barcelona, Spain; 3 Institute of Protein Research, Russian Academy of Sciences, Pushchino, Russia

## Abstract

**Background:**

Divergence of two independently evolving sequences that originated from a common ancestor can be described by two parameters, the asymptotic level of divergence *E *and the rate *r *at which this level of divergence is approached. Constant negative selection impedes allele replacements and, therefore, is routinely assumed to decelerate sequence divergence. However, its impact on *E *and on *r *has not been formally investigated.

**Results:**

Strong selection that favors only one allele can make *E *arbitrarily small and *r *arbitrarily large. In contrast, in the case of 4 possible alleles and equal mutation rates, the lowest value of *r*, attained when two alleles confer equal fitnesses and the other two are strongly deleterious, is only two times lower than its value under selective neutrality.

**Conclusions:**

Constant selection can strongly constrain the level of sequence divergence, but cannot reduce substantially the rate at which this level is approached. In particular, under any constant selection the divergence of sequences that accumulated one substitution per neutral site since their origin from the common ancestor must already constitute at least one half of the asymptotic divergence at sites under such selection.

**Reviewers:**

This article was reviewed by Drs. Nicolas Galtier, Sergei Maslov, and Nick Grishin.

## Background

Constant selection mostly manifests itself as negative selection, because permanently beneficial alleles tend to become common. Negative selection constrains and decelerates evolution. Slower evolution of more functionally important nucleotide sites, such as nonsynonymous coding sites, compared to less important sites, such as synonymous sites, is one of the most pervasive patterns in evolution at the sequence level and reflects ubiquity of negative selection and relative rarity of positive selection. Constant selection can accelerate evolution when it favors more mutable alleles [1], but the magnitude of this effect is substantial only when a number of conditions are met [2].

Still, the impact of constant selection on the rate of evolution, manifested in the dynamics of divergence of two independently evolving sequences that originated from a common ancestor, is not as simple as one might think, and should be viewed from two complementary perspectives. Indeed, constant selection affects both 1) the asymptotic value of sequence divergence *E*, reached by two sequences after a long time, and 2) the rate *r *at which the divergence between two sequences approaches *E*. Here, we separately study the impact of constant selection on *E *and on *r*.

## Model

Consider one locus (site) with *I *alleles *A*_1_, ... *A*_*I *_with frequencies *x*_1_, ..., *x*_*I*_, and fitnesses *1, 1-s*_2_, ... *1-s*_*I*_, where *s*_*i *_are coefficients of selection (without loss of generality, the fitness of one of the alleles can be assumed to be 1). A population is either haploid or diploid with intermediate dominance. The rate of mutation of the *i*th allele into the *j*th allele is *μ*_*i*, *j*_, and the effective size of the population is *N*_*e*_. We assume that *N*_*e*_*μ*_*i*, *j*_*<<*1 for all *i*, *j*, so that most of the time one of the alleles is fixed in the population [3], and the dynamics of the model consists of allele replacements occurring at a site at random moments. We consider dynamics of divergence of two long sequences of sites, all with the same properties, that were identical at the moment when they originated from their last common ancestor and evolved independently after this moment. The level of divergence *D *will be characterized by the fraction of homologous sites occupied by different alleles.

## Results

### Asymptotic value of divergence *E*

Obviously, after enough time passes since the moment when two sequences begun to diverge, all traces of over-random similarity between them are lost, and *D *reaches its asymptotic value *E*, given by(1)

where *X*_*i *_is the equilibrium value of *x*_*i *_under given mutation and selection. *X*_*i *_can be found from a set of *I *linear equations (eq. 10 from [3]; eq. (11) below presents this set in the case of *I *= 4). A strong enough constant selection favoring one allele over all others can make *E *arbitrarily close to 0.

### The rate r of approaching E: selective neutrality

Let us now concentrate on how *D *approaches *E*. We will characterize the rate of this approach by *r*, the power of an exponential decline of the deviation of *D *from *E*, and, equivalently, by the half-approach period *τ *= *ln2/r*. Indeed, we will see that *D *approached *E *exactly (in special cases) or approximately (in the general case) exponentially.

Let us first consider the case of no selection: *s*_*i *_= 0 for all *i*. Let us also assume that *μ*_*i*, *j *_= *μ *for all *i*, *j*. Then,(2)

because the probability of a mismatch becoming a match during a short interval of time *dt *is *2 μdt*, and the probability of a match becoming a mismatch is *2(I-1)μdt *(see Chapters 3 and 4 from [4]). The solution of this equation with *D *= 0 at time 0 can be obtained from(3)

and is given by(4)

**Figure 1:**
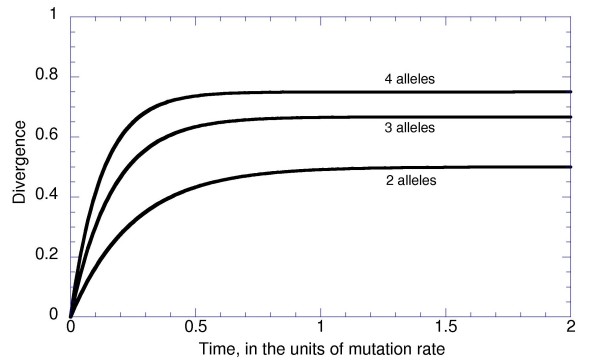
**Dynamics of divergence of independently evolving sequences of 2-, 3-, and 4- allelic loci without selection**.

Thus, *E *= 1/2, 2/3, and 3/4; and *r *= *4 μ*, *6 μ*, and *8 μ*, with 2, 3, and 4 selectively neutral alleles (Figure 1). We will mostly assume that our loci are nucleotide sites, so that these three cases are particularly relevant. Note, that nucleotide sites where strong selection acts against one or two "forbidden" nucleotides but fitnesses of the remaining three or two "permitted" nucleotides are the same are described by the model with 3 or 2 selectively neutral alleles.

### The rate r of approaching *E*: two alleles with selection

Let us now consider the case of only two alleles *A*_1 _and *A*_2_, under an arbitrary selection, described by *s*_2 _= *s*. We will keep the assumption that *μ*_*i*, *j *_= *μ *for all *i*, *j*, which in this case simply means that the rates of reciprocal mutations are the same. The dynamics of *D *are described by (5)

where *x*_*i*, *j *_is the frequency of pairs of sites occupied by allele *A*_*i *_in one sequence and allele *A*_*j *_in the other sequence (due to symmetry of the two sequences, we will use this notation assuming that *i *≤ *j*), and *f*_*i*, *j *_is the rate at which allele *i *is replaced with allele *j*. Then, *D = x*_1,2 _and *x*_1,1 _= *x*_1 _- 0.5*D*, because *x*_1 _= 0.5*x*_1,2_*+ x*_1,1_. Analogously, *x*_2,2 _= 1-*x*_1 _- 0.5*D*, because *x*_2_*+ x*_1 _= 1.

Let us assume that the ancestral sequence from which the two sequences under consideration begun their independent divergence had already attained equilibrium allele composition. Then, *x*_1 _= *X*_1 _is invariant and is equal to *e*^*S*^/*(e*^*S*^*+ *1) (eq. 6 from [3]). According to eq. 7 from [3], *f*_1,2 _= -*μS/(*1-*e*^*S*^) and *f*_2,1 _= *μS/(*1-*e*^-*S*^), where *S = 4N*_*e*_*s*, in the case of diploidy. Thus, the equation (5) can be written as:(5)

with a solution:(6)

Thus,(7)

and(8)

**Figure 2:**
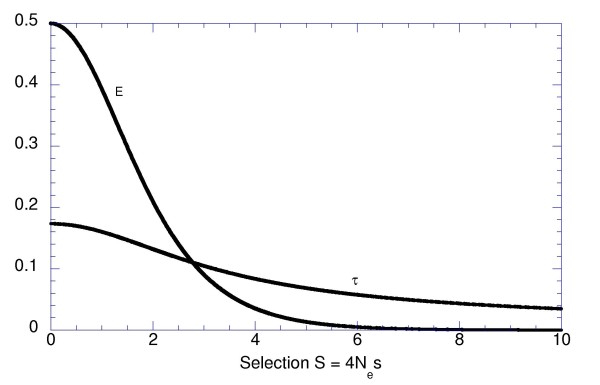
**Asymptotic divergence *E *and half-approach period *τ = ln *2/*r *in the case of two alleles, as functions of the selection coefficient**.

It is easy to show that *r(S) *has a single minimum at *S *= 0. Therefore, with only two permitted alleles the lowest rate *r(0) *= 4 *μ *of approaching E, and the longest half-approach period *τ = ln*2/*(*4*μ) *is achieved under neutrality (4), where the value of *E *= 1/2 is the highest, because *X*_1 _= *X*_2_. When *S *increases, *E *rapidly approaches zero, and *r(S) *slowly increases without a limit (Figure 2). A more general case of unequal rates of reciprocal mutations can also be considered using eq. 7 from [3].

### The rate r of approaching E: general 4-allele case

Let us now consider the general case with *I *= 4. Here it will be more convenient to use letters *A*, *T*, *G*, and *C *to denote the four alleles. With 4 alleles under selection, *D *becomes dynamically insufficient, and the divergence of sequences needs to be described in terms of frequencies of the six possible mismatches *x*_*i*, *j *_([5], eq. 1):(9)

where the rates of allele replacements *f*_*i*, *j*_, as before are given by eq. 7 from [3] with the coefficient of selection reflecting the difference between fitnesses of the *i*th and the *j*th allele and *f*_*i*, *noti *_is the sum of rates of replacement of *i*th allele into all other alleles. Frequencies of the four matches are inferred from the dynamical variables: *x*_*A*, *A *_= *X*_*A *_- 0.5*(x*_*A*, *T*_*+ x*_*A*, *G*_*+ x*_*A*, *C*_); *x*_*T*, *T *_= *X*_*T *_- 0.5*(x*_*A*, *T*_*+ x*_*T*, *G*_*+ x*_*T*, *C*_); *x*_*G*, *G *_= *X*_*G *_- 0.5*(x*_*A*, *G*_*+ x*_*T*, *G*_*+ x*_*G*, *C*_); *x*_*C*, *C *_= *X*_*C *_- 0.5*(x*_*A*, *C*_*+ x*_*T*, *C*_*+ x*_*G*, *C*_), where *X*_*i*_, are obtainable from [3] (eq 10)(10)

under the assumption that *x*_*A*_*+ x*_*T*_*+ x*_*G*_*+ x*_*C *_= 1. *D *is the sum of all the six variables in (10).

**Figure 3:**
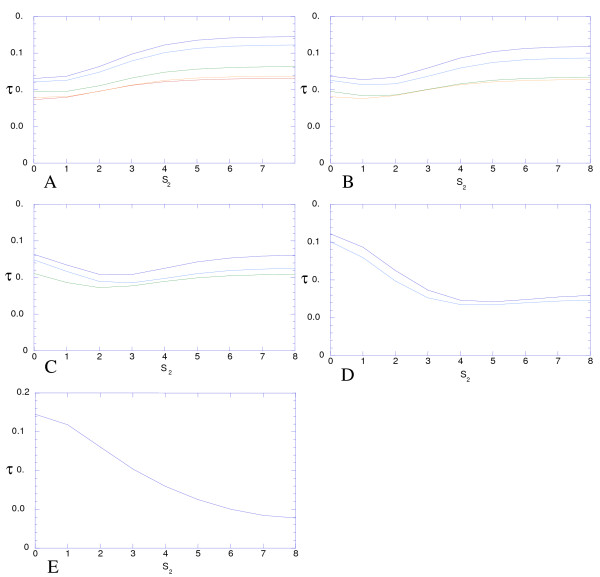
**Half-approach period *τ = ln *2/*r*, in the units of the mutation rate, in the case of four alleles, under different modes of selection**. *S*_3 _= 0, 1, 2, 4, and 8 (A - E). *S*_4 _= 0, 1, 2, 4, and 8 (red, orange, green, light blue, deep blue).

**Figure 4:**
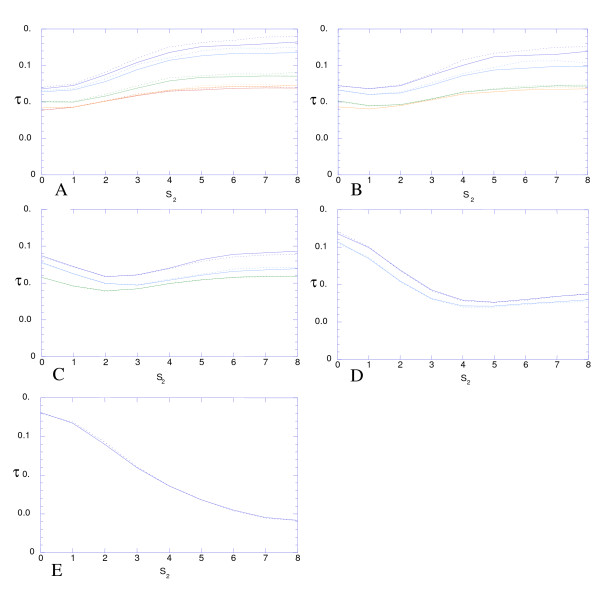
**The same as Figure 3, but, for each mode of selection, the half-approach period presented in the average of its values obtained under 1000 randomly generated matrices of *μ*_*i*, *j*_, in which values of *μ*_*i*, *j *_were drawn independently from a uniform distribution that varied between 0.5 and 1.5 and the matrices were symmetric (solid lines) or arbitrary (dotted lines)**.

Numerical investigation of (10) revealed the following facts:

1) *D *approaches *E *strictly exponentially only in the case of just two permitted alleles. Otherwise the approach is decelerating: the time it takes for *D *to decline from 0 to *E*/2, *t*_0,1/2 _is shorter than the time *t*_1/2,3/4 _during which D declines from *E*/2 to 3*E*/4. However, this effect is weak: *t*_1/2,3/4 _never exceeds *t*_0,1/2 _by more than 10%. Thus, we can safely regard *t*_0,1/2 _as the half-approach period *τ*.

2) *D *approaches *E *at a rate that cannot be below *r *= 4 *μ*, which is only 2 times less than *r *= 8 *μ*, the rate for the case of 4 neutral alleles (*S*_2 _= *S*_3 _= *S*_4 _= 0) (eq 4). This lowest *r *= 4 *μ *was observed only in the case of 2 neutral alleles (*S*_2 _= *0; S*_3 _= *S*_4_-*> 8*) (eq 9). In contrast, *r *can be arbitrarily large, but this happens only when just one allele is strongly favored (*S*_2_, *S*_3_, *S*_4_-*> 8*) (eq 9). When selection is moderate (*S *≤ 2), 4 *μ < r <*8 *μ *(Figure 3).

3) If the assumption of equal mutation rates is relaxed, the expected value of *r *under a particular mode of selection does not change much (Figure 4).

## Discussion

We have found a contrast between how constant selection affects the asymptotic level of divergence of independently evolving sequences *E *and the rate *r *at which this level is approached. While *E *always declines and can be affected arbitrarily strongly, *r *can both increase and decrease. The increase of *r *can be arbitrarily large, and occurs under conditions when *E *approaches 0, *i. e*. when selection strongly favors one allele over all others. However, *r *cannot decline by more than a factor of two (assuming equal rates of all mutations) relative to the case of 4 selectively neutral alleles, with the lowest *r *attained when two alleles have equal high fitnesses and the other alleles are strongly selected against. Unequal mutation rates apparently do not affect this conclusion. Of course, if selection coincidently permits only those two alleles that rarely mutate into each other, it can lead to an arbitrary decrease of *r*. However, in a general situation, unequal mutation rates do not substantially affect the impact of selection on *r*.

In other words, the highest, for the case of 4 alleles, asymptotic divergence *E *= 3/4 is approached with a half-approach period *τ = ln*2/*(*8 *μ) *≈ 0.087 *μ*^-1^, which is neither the shortest nor the longest possible under constant selection. However, the longest possible half-approach period *τ*_*max *_= *ln*2/(4 *μ) ≈ *0.173 *μ*^-1^, is just two times longer. Thus, the assertion that constant selection impedes evolution mostly describes its impact on the asymptotic level of divergence, and not on the rate at which this level is attained.

After *D *travels half of its way to *E *in the case of 4 neutral alleles, *D *= 3/8. For this *D*, application of Jukes-Cantor formula [4], (eq. 4.2) produces the following value of the per-site number of nucleotide substitutions per a selectively neutral site:(11)

Indeed, under selective neutrality the rate of evolution is equal to mutation rate, so that *K*_1/2, *neutral *_= 6 *μτ*_*neutral *_with *I *= 4, and *τ*_*neutral *_= *ln*2/(8 *μ) *in this case. Thus, because constant selection cannot reduce the rate of approaching E by more that a factor of 2, at all kinds of sites under constant selection divergence between two species with *K*_*neutral *_= 1.04 must already represent, at the very least, 50% of their equilibrium divergence, as long as the allelic composition of the diverging sequences was at equilibrium from the very beginning. And divergence of all orthologous sequences that are under constant selection in species with *K*_*neutral *_*> *5 must be already very close to its asymptotic level.

It did not escape our attention that the dynamics of divergence of functionally important sequences are usually inconsistent with this key property of the model considered here: orthologous sequences keep diverging even between very evolutionarily remote genomes. There would be little hope to reconstruct the history of a molecule from extant sequences if selection coefficients were constant in time. In this case, sequences having diverged long enough would essentially include only invariant (strongly selected) and saturated (weakly selected) sites. Since we know that protein sequences can actually help in measuring divergences and building deep phylogenies, this suggests that variations in time of N_*e *_and/or of selection coefficients at individual sites are common, and that their rate of change is what controls in the first place the amount of amino-acid differences between distantly related extant sequences.

## Competing interests

The authors declare that they have no competing interests.

## Authors' contributions

FAK designed this study, ASK formulated the theory and performed most of the computational analyses, ISP derived the analytical solution, DNI participated in finding the analytical solution and in performing additional computational analyses. All authors read and approved the final manuscript.

## Reviewers' comments

### Reviewer 1

Referee 1:

Nicolas Galtier

CNRS-Université Montpellier II

Montpellier, France

This manuscript implements a very good idea, namely injecting population genetic parameters, typically used to model microevolution, in substitution matrices, typically used to model long-term sequence evolution. The two must be connected in principle, yet classical Markov models used in phylogenetics are made of arbitrary parameters.

These theoretical results are good to know and keep in mind, even though they perhaps do not apply so frequently to real data, as correctly acknowledged, given the assumptions of constant in time population size and selection coefficients. At any rate, this piece might stimulate other studies trying to understand long-term sequence evolution in the light of population genetics. Here are a couple of more specific comments.

1. I am not sure if the model is haploid or diploid. Diploidy is apparently assumed at some stage, but since there is no description of the fitness of a diploid genotype, I guess codominance is assumed. In this case, the diploid model is equivalent to the haploid one, so I would suggest to keep it haploid throughout for simplicity. Or if you make it diploid, please discuss dominance effects.

Indeed, this needed to be clarified. Bulmer's framework we used can handle either haploidy or diploidy with codonimance. We assumed the latter case, but the only difference this made is that *S = 4N*_*e *_*s*, instead of *S = 2N*_*e *_*s *with haploidy. This is now stated.

2. I feel like a better job could be made in accounting for, and referring to, the 80's/90's literature on Markov models for pairwise distance calculation between sequences. The papers by Rodriguez et al [5] and Lockhart et al [6], for instance, include equations very close to equation 10 of this manuscript. The matrix expressions and calculations used in these papers might help, by the way - I suspect that the numerical exploration of equation 10 performed by the authors could be done analytically. Once you have expressed the substitution rate from allele i to allele j in terms of mutation, selection and drift, you are within Rodriguez's formalism.

**Indeed, we needed to cite Rodriguez et al.**[5], **because our eq. 10 is essentially identical to theirs basic equation 1. However, as Dr. Galtier noted, Rodriguez *et al*. **[5]**did not study the dependence of solution to this equation on mutation and selection parameters separately; instead they only considered fluxes of allele substitutions. In contrast, we do not see how our results are related to that of Lockhart *et al*.**[6], **because they were primarily concerned with taking into account different nucleotide compositions of different sequences, and we, as well as Rodriguez *et al*.**[6]**, assumed these to be invariant. Of course, our eq. 10 can, as a system of linear ordinary differential equation, be solved analytically using matrix exponents. However, it will probably still be necessary to investigate this solution numerically, to understand its properties.**

3. If I understand correctly, this manuscript demonstrates that there would be little hope to reconstruct the history of a molecule from extant sequences if selection coefficients were constant in time. In this case, sequences having diverged long enough would essentially include only invariant (strongly selected) and saturated (weakly selected) sites. Since we know that protein sequences can actually help in measuring divergences and building deep phylogenies, this suggests that variations in time of Ne and s are common, and that their rate of change is what controls in the first place the amount of amino-acid differences between extant sequences - at least as far as nonadaptive changes are concerned. This conclusion is reminiscent of Gillespie's thoughts about the overdispersed molecular clock. It makes the more or less clock-like behavior of many known molecules even more amazing to me.

We are very thankful for this interpretation of our main conclusion, and we plagiarize it for Discussion with minor changes, placing it at the very end of the paper. In contrast, we do not see a direct correspondence between our results and Gillespie's overdispersed molecular clock. Indeed, changes of selection, which can lead to prolonged divergence, do not necessarily need to occur at random moments of time.

### Reviewer 2

Referee 2:

Sergei Maslov

Brookhaven National Laboratory Upton, NY, USA

The conclusions of this study can be summarized in the following statement: the strength of selection has a relatively small effect of the rate at which the evolutionary steady state is approached. Authors offer detailed derivations for the case of a locus consisting of a single nucleotide site. Hence their number of alleles is limited to 4 (A, C, G, and T). In cases where the selection indeed operates at the level of a single nucleotide the derivation presented in the manuscript is through and exhaustive. The mathematically transparent summary of the central results is contained in Eqs. (8) and (9).

I found the discussion part of this manuscript to be somewhat cryptic. For example, I didn't get the point authors were trying to make by using the celebrated Watson and Crick opening sentence in: "It did not escape our attention that the dynamics of divergence of functionally important sequences are usually inconsistent with this key property of the model considered here". What key property of their model authors are talking about?

We expanded this place a bit, using insightful comments of Dr. Galtier. We believe that the main reason why our analysis may be interesting is that it provides a proof by contradiction to the assertion that selection at individual sites keeps constantly changing, which has long been suspected but never demonstrated convincingly. However, this is a separate story.

Another confusing part of the discussion is that after deriving K_1/2,neutral = 0.52 in Eq. (12) the authors state that sequences diverged to the level of

K_neutral = 2K_1/2,neutral = 1.04 represent at least 50% of their equilibrium divergence. I thought that is exactly how K_1/2,neutral was defined. Why double it? In short, I feel the manuscript would benefit from giving a bit more details in the discussion session.

Double it because constant selection can, in the extreme situation, half the rate at which the asymptotic level of divergence is approached. This is now explained.

Another thing, I would like authors to better describe what practical questions their mathematical observations help to understand? I am shooting in the dark here but do these results might explain a puzzlingly uniform rate at which non-neutral substitutions accumulate in the long-term evolutionary experiment by Lenski and collaborators [7]? Having a few concrete examples where the mathematical treatment presented in the manuscript is important would greatly improve the readability of the manuscript.

One cannot be sure that Lenski's lab data are relevant to our analysis.  Indeed, they placed their bacteria in a novel environment, so their system reflects a dynamics of adaptation and may not be at equilibrium, in contrast to our assumptions.  We believe that our analysis is important because it demonstrates that, in the long term, evolution of even conservative orthologs does not proceed under constant selection. 

### Reviewer 3

Referee 3:

Nick Grishin

University of Texas Southwestern Medical Center

Dallas, TX, USA

This reviewer provided no comments for publication.
